# 157. Implementation of multidisciplinary diagnostic stewardship for *Clostridioides difficile* infection during the COVID-19 pandemic at a small Japanese hospital

**DOI:** 10.1093/ofid/ofad500.230

**Published:** 2023-11-27

**Authors:** Yasuhiro Sasaki, Masataka Yano, Ayumi Umehara, Yasuaki Tagashira

**Affiliations:** Tama-Nambu Chiiki Hospital, Tama-city, Tokyo, Japan; Tama-Nambu Chiiki Hospital, Tama-city, Tokyo, Japan; Tama-Nambu Chiiki Hospital, Tama-city, Tokyo, Japan; Tokyo Medical and Dental University, Bunkyo-ku, Tokyo, Japan

## Abstract

**Background:**

*Clostridioides difficile* infection (CDI) is a common, healthcare-associated infection. However, its incidence has reportedly declined in the United States owing to the COVID-19 pandemic. On the other hand in Japan, testing for CDI has been infrequent, suggesting that this disease may be underdiagnosed. The present study examined the implementation of multifaceted, diagnostic stewardship for CDI in a small Japanese hospital during the pandemic.

**Methods:**

The following interventions were introduced at Tama-Nambu Chiiki Hospital, a small community hospital in Tokyo: 1) criteria for CDI testing were added to the hospital's guidelines; 2) clinical pharmacists and nurses assessed the need for CDI testing in patients with nosocomial diarrhea and made recommendations for CDI testing to physicians; 3) a tutorial on CDI testing was given to physicians, nurses, and pharmacists; 4) data on the density of CDI testing and the CDI incidence in the hospital were provided to healthcare workers every three months. The outcomes were CDI testing frequency and the CDI incidence per 10,000 patient days. Segmented regression in interrupted time-series analysis (ITSA) was performed before the pandemic (2018/04-2020/03), after the pandemic (2020/04-2022/03), and after the intervention (2022/04-2023/03).

**Results:**

The frequency of CDI testing tended to increase before the pandemic (+0.16/10,000 PD, P=0.28) but decreased significantly after the pandemic (-0.79/10,000 PD, P=0.02), then increased significantly immediately after the intervention (+29.6/10,000 PD, P< 0.01). Similarly, the incidence of CDI increased significantly before the pandemic (+0.23/10,000PD, P=0.02) but decreased significantly after the pandemic (-0.45/10,000PD, P=0.01). Implementation of the intervention resulted in an immediate, significant increase in the CDI incidence (+6.8/10,000PD, P< 0.01).

**Conclusion:**

Multifaceted, diagnostic stewardship involving multiple specialists was effective in improving CDI testing, suggesting that appropriate testing can contribute to the accurate diagnosis of CDI.

**Disclosures:**

**All Authors**: No reported disclosures

